# Derivative component analysis for mass spectral serum proteomic profiles

**DOI:** 10.1186/1755-8794-7-S1-S5

**Published:** 2014-05-08

**Authors:** Henry Han

**Affiliations:** 1 Department of Computer and Information Science, Fordham University, New York NY 10458 USA; 2 Quantitative Proteomics Center, Columbia University, New York 10027 USA

## Abstract

**Background:**

As a promising way to transform medicine, mass spectrometry based proteomics technologies have seen a great progress in identifying disease biomarkers for clinical diagnosis and prognosis. However, there is a lack of effective feature selection methods that are able to capture essential data behaviors to achieve clinical level disease diagnosis. Moreover, it faces a challenge from data reproducibility, which means that no two independent studies have been found to produce same proteomic patterns. Such reproducibility issue causes the identified biomarker patterns to lose repeatability and prevents it from real clinical usage.

**Methods:**

In this work, we propose a novel machine-learning algorithm: derivative component analysis (DCA) for high-dimensional mass spectral proteomic profiles. As an implicit feature selection algorithm, derivative component analysis examines input proteomics data in a multi-resolution approach by seeking its derivatives to capture latent data characteristics and conduct de-noising. We further demonstrate DCA's advantages in disease diagnosis by viewing input proteomics data as a profile biomarker via integrating it with support vector machines to tackle the reproducibility issue, besides comparing it with state-of-the-art peers.

**Results:**

Our results show that high-dimensional proteomics data are actually linearly separable under proposed derivative component analysis (DCA). As a novel multi-resolution feature selection algorithm, DCA not only overcomes the weakness of the traditional methods in subtle data behavior discovery, but also suggests an effective resolution to overcoming proteomics data's reproducibility problem and provides new techniques and insights in translational bioinformatics and machine learning. The DCA-based profile biomarker diagnosis makes clinical level diagnostic performances reproducible across different proteomic data, which is more robust and systematic than the existing biomarker discovery based diagnosis.

**Conclusions:**

Our findings demonstrate the feasibility and power of the proposed DCA-based profile biomarker diagnosis in achieving high sensitivity and conquering the data reproducibility issue in serum proteomics. Furthermore, our proposed derivative component analysis suggests the subtle data characteristics gleaning and de-noising are essential in separating true signals from red herrings for high-dimensional proteomic profiles, which can be more important than the conventional feature selection or dimension reduction. In particular, our profile biomarker diagnosis can be generalized to other omics data for derivative component analysis (DCA)'s nature of generic data analysis.

## Background

With the surge in serum proteomics, large volumes of mass spectral serum proteomic data are available to make molecular diagnosis of complex disease phenotypes possible. As a promising way to revolutionize medicine, serum proteomics demonstrates a great potential in identifying novel biomarker patterns from the serum proteome for diagnosis, prognosis, and early disease discovery [1-3]. However, high-performance disease phenotype discrimination remains a challenge in translational bioinformatics due to special characteristics of serum proteomics data, in addition to its well-known data reproducibility issue, which means that no two independent studies have been found to produce same proteomic patterns [3-5].

A serum proteomic data set can be represented as a matrix $X\in {ℜ}^{n\times p}$ after preprocessing, where each row represents protein expression at a mass-to-charge (*m/z*) ratio of peptides or proteins and each column represents protein expression from a sample/observation (e.g., a control or cancer subject) across all *m/z *ratios in experiment. The number of rows is much greater than the number of columns, p<<n, that is #variables (peptides/proteins) is much greater than #samples. Usually $n\sim O\left(10^{4}\right),$ and $p\sim O\left(10^{2}\right).$ Although there are a large amount of *m/z *ratios (peptides or proteins), only a few numbers of them (e.g., peaks) have meaningful contribution to disease diagnosis and data variations. Moreover, such data are not noise-free because normalization methods cannot remove built-in systems noise from mass spectrometry technology itself [6,7]. In particular, the high-dimensionality directly prevents conventional classification algorithms from achieving clinical rivaling disease diagnosis, limits its generalization capability or even causes some regularity problem in classification [7].

Quite a lot feature selection methods have been employed in serum proteomic data classification to glean informative features, reduce dimension, or conduct de-noising in order to achieve high accuracy disease diagnosis [7-10]. It is noted that a feature refers to a row in a serum proteomic data set, which are biologically peptides or proteins. In this work, we categorize them into input-space and subspace methods respectively. The former seeks a feature subset $X^{\prime }\in {ℜ}^{m\times p},$m<<n in the same space ${ℜ}^{n\times p}$ as input data  X by conducting a hypothesis test (e.g., *t-test*), or wrapping a classifier to features recursively; The latter conducts dimension reduction by transforming data  X into a subspace *S *induced by a linear or nonlinear transformation f:X→S where $S=span\left(s_{1},s_{2}\ldots s_{k}\right),$$s_{k}\in {ℜ}^{n\times p},$k≤p≤n, and seeking meaningful linear combinations of features. For example, the subspace spanned by all principal components when the transformation is induced by principal component analysis (PCA) [11].

All subspace methods can be formulated as a matrix decomposition problem: $X\sim SP^{T},$$S\in {ℜ}^{n\times k},$$P\in {ℜ}^{p\times k}$ where different methods construct different basis matrices *S *and different feature matrices *P *according to different termination conditions. For instance, nonnegative matrix factorization (NMF) seeks nonnegative matrix decomposition such that $\left||X\sim SP^{T}|\right|$ is minimized under an Euclidean distance or K-L divergence [12,13]. In fact, almost all PCA, ICA, and NMF 's extensions such as nonnegative principal component analysis (NPCA), sparse NMF, and other methods such as random projection methods all fall into this category [8,12-16].

However, these methods may not always contribute to improving diagnosis in serum proteomics robustly. Instead, it was reported that classifiers integrated with them may usually demonstrate large oscillations in performance for different data sets and some even got worse performance than the case without feature selection [7,8,10]. Moreover, there was no systematic work on addressing the limitations of those feature selection methods. In this work, we address these methods' limitations before introducing our novel derivative component analysis (DCA).

### Lack of de-noising schemes

The input-space methods usually lack de-noising schemes and assume input data is clean or nearly clean. Such an assumption can be true for the data that are by nature clean or with quite low-level noise (e.g., financial data). However, it appears to be inappropriate for serum proteomics data since they usually contain nonlinear noise from profiling systems, and technical/biological artifacts. The noise would enter feature selection as outliers and produce less informative or even ad-hoc feature sets (e.g., peaks with less biological meaning), which would lead to an inaccurate or even poor decision function in classification and affect the disease phenotype diagnosis, generalization, and biomarker discovery in translational bioinformatics.

### Latent data characteristics missing

Those subspace methods have difficulties in capturing subtle or latent data characteristics, because subspace methods transform data into another subspace to seek meaningful feature combination and original spatial coordinates are 'lost', which makes it almost impossible to track those features contributing to the behaviors. The latent data characteristics refer to subtle data behaviors interpreting transient data changes (we use words 'subtle' and 'latent' equivalently when describing data characteristics in our context). Quite different from global data characteristics that referring to the holistic data behaviors interpreting long-time interval data changes, subtle data characteristics have to be represented by the first or even high-level derivative of data mathematically [8,10].

We use principal component analysis (PCA) as an example to address this issue. Given input data with zero mean $X\in {ℜ}^{n\times p},$ the subspace is spanned by selected PCs, i.e. $S=span\left(u_{1},u_{2},\ldots u_{k}\right)$1≤k≤p. Since each subspace basis (PC) receives contributions from all features (peptides/proteins) in the linear combinations, changes in one feature will inevitably affect all bases globally. Although it is biologically important to identity which protein/peptide has more contributions to the data change, it is quite hard to achieve it because their coefficients in the linear combination are not usually comparable [6]. Moreover, subspace basis calculation does not involve the feature derivative information or its related approximation, which causes each PC not to be able to capture latent (subtle) data characteristics well. As such, only global data characteristics can be captured well and subtle data characteristics, which are essential in achieving high performance diagnosis, may be totally missed. For example, some malignant and benign tumors may have similar global data characteristics but different subtle data characteristics in serum profiling. As such, detecting subtle data characteristics is essential to achieve a clinical level diagnosis.

Although various subspace methods such as sparse-PCA, nonnegative-PCA, and sparse-NMF [12,8,14,16], have been proposed to enhance subtle data characteristics capturing by imposing non-negativity or sparsity constraints in order to seek subspace bases through solving a nonlinear optimization problem, they are usually characterized by high complexities (e.g., nonnegative PCA [6,8]) and none of them seems to be able to catch subtle data characteristics by examining the features 'beyond' their original data level.

In this work, we propose a *de novo *derivative component analysis (DCA), which evolves from author's previous work in gene and protein expression omics data analysis [8,9], to overcome the current feature selection methods' weaknesses for the sake of clinical level disease diagnosis in serum proteomics. It is worthwhile to point out that our DCA is a novel machine learning algorithm based on our global and local feature selection theory proposed in [8], which is more complicated and powerful than the serum proteomics data analysis methods that straight-forwardly apply wavelet transforms to a proteomic sample and conduct classic statistical tests to following wavelet coefficients [17]. Our DCA employs discrete wavelet transforms (DWT) [18] to look at serum proteomics data in *'multiple windows*' to extract latent data characteristics and achieve de-noising by retrieving 'data derivatives'.

Furthermore, we employ benchmark serum proteomic data to demonstrate DCA's superiority in disease diagnosis by proposing a novel diagnosis algorithm DCA-SVM and comparing it with the other state-of-the-art peers. The exceptional performance of our DCA-SVM suggests it can be a potential way to overcome the serum proteomics' reproducibility by viewing input data as a profile biomarker. As a key result in this work, we present DCA-MARK, a DCA-based biomarker discovery algorithm that strongly demonstrates high-dimensional serum proteomics data's linear separability, which not only has an important meaning in machine learning, but also has practical impacts on translational bioinformatics for its novelty. To the best of our knowledge, it is the first work that is able to linearly separate high-dimensional serum proteomic data with few biomarkers.

## Derivative Component Analysis (DCA)

Different from its conventional definition, a feature is no longer viewed as an indecomposable information unit in DCA. Instead, all features are hierarchically decomposed into different components to discover data derivatives to capture subtle data characteristics and conduct de-noising. The proposed derivative component analysis (DCA) consists of the following three steps.

First, a discrete wavelet transform (DWT) is applied to all features to decompose it hierarchically as a set of detail coefficient matrices $cD_{1},cD_{2}\ldots cD_{J}$ and an approximation matrix $cA_{J}$ under a transform level *J*. Since DWT is done on a set of dyadic grid points hierarchically, the dimensionalities of the approximation and detail coefficient matrices shrink dyadically from level 1 to level *J *[17]. For example, given a proteomic data set with 10 samples across 1024 *m/z *ratios under a DWT with a transform level *J = 5, cD_1 _*is a 10 × 512 matrix and *cD_2 _*is 10 × 256 matrix. Similarly, *cD_5 _*and *cA_5 _*both are 10 × 32 matrices.

The approximation matrix and coarse level detail coefficient matrices (e.g., *cDJ*) capture the global data characteristics, because they contain contributions from the features disclose slow changes in 'long-time windows', if we view each *m/z *ratio as a corresponding time point in our context. Similarly, the fine level detail coefficient matrices (e.g., *cD_1_, cD_2_*), capture subtle data characteristics, because they contain contributions from the features that disclose quick changes in 'short-time windows'. In fact, the fine level detail matrices are components to reflect data derivatives in different time windows. Furthermore, most system noises are hidden in these components for its heterogeneity with respect to true signals. In summary, the first step separates global characteristics, subtle data characteristics, and noise in different resolutions.

Second, retrieve the most important subtle data behaviors and remove noise by reconstructing the fine level detail coefficient matrices before or at a presetting cutoff level *τ *(e.g*.,τ = 3*). Such construction consist of two steps: 1) Conduct principal component analysis (PCA) for the detail matrices $cD_{1},cD_{2}\ldots cD_{\tau }$ 2) Reconstruct each detail coefficient matrix by using its first *m *leading loading vectors, i.e., principal components, in its each principal component (PC) matrix. Usually, we set *m *= 1, i.e., we employ the first principal component to reconstruct each detail coefficient matrix, which means we only retrieve the most important subtle data characteristics in detail coefficient matrix reconstruction. In fact, the first PC based reconstruction also achieves de-noising by suppressing noise's contribution in the detail coefficient matrix reconstruction because noise has is usually unlikely to appear in the 1^st ^PC.

On the other hand, the coarse level detail coefficient matrices after the cutoff *τ *: $cD_{\tau +1},\;cD_{\tau +2}\ldots cD_{J}$ and approximation coefficient matrix $cA_{J}$ are kept intact to retrieve global data characteristics. In fact, parameter *m *can be also determined by using a variability explanation ratio $\rho_{m}$ defined as follows, such that it is greater than a threshold  ρ (e.g., ρ=60%), which is the variability explanation ratio by the first principal component of those detail coefficient matrices before or equal the cutoff.

### Variability explanation ratio

Given a data set with *n *variables and *p *observations, usually, *p < n*, the variability explanation ratio is the ratio between the variance explained by the first *m *PCs and the total data variances: $\rho_{m}=\overset{m}{\underset{i=1}{\sum }}\sigma_{i}/\overset{p}{\underset{i=1}{\sum }}\sigma_{i}$, where $\sigma_{j}$ is the variance explained by the $j^{th}$ PC, which is actually the $j^{th}$ eigenvalue of the covariance matrix of the input proteomic data.

It is noted that such a selective reconstruction process in the second step extracts the most important subtle data characteristics and conduct de-noising by suppressing the contribution from system noise. This is because only one or few principal components are employed in reconstructing each targeted fine level coefficient matrix $cD_{j}$ and those less important and noise-contained principal components are dropped in reconstruction.

Third, conduct the corresponding inverse DWT by using the current detail and approximation coefficient matrices to obtain an meta-data $X_{*}$ that is the corresponding de-noised data set with subtle data characteristics extraction and system noise removal, because of the highlight of the most significant subtle data behaviors in the "derivative components" based reconstructions. The meta-data are just 'true signals' separated from red herrings that share the same dimensionality with the original data but with less memory storage because less important PCs are dropped in our reconstruction.

It is noted that, unlike traditional feature selection methods, DCA is an implicit feature selection method, where useful characteristics are selected implicitly without an obvious variable removal or dimension reduction. Algorithm 1 gives the details about DCA as follows, where we use $X^{T}$ instead of *X *to represent input proteomic data for the convenience of description, i.e. each row is a sample and each column is a feature in the current context.

Algorithm Derivative Component Analysis (DCA)

1. **Input: **$X^{T}=\left[x_{1},x_{2},\ldots x_{n}\right]$$x_{i}\in {ℜ}^{p},$*DWT level J; cutoff τ; wavelet *ψ,*thereshold *ρ,

2. **Output: **Meta-data $X_{*}^{T}$

3. **Step 1**. *Column-wise discrete wavelet transforms (DWT)*

4.     Conduct J-level DWT with wavelet  ψ for each column of $X^{T}$ to obtain

      $\left[cD_{1},\;cD_{2}\ldots cD_{J};cA_{J}\right],$$cDj\in {ℜ}^{p_{j}\times n},$$cA_{J}\in {ℜ}^{p_{J}\times n},$ and $p_{j}=\left[p/2^{j}\right],$j=1,2,…J.

5. **Step 2. ***Derivative component analysis for latent data characteristics extraction and de-noising*

6. *for j *= 1 to *J*

7.   if *j *≥ *τ*

8.     a) Do principal component analysis for each detail matrix $cD_{j}$ to obtain its PC and score matrix,

9.         $U=\left[u_{1},u_{2},\ldots u_{p}\right],$$u_{i}\in {ℜ}^{n}$ and $S=\left[s_{1},s_{2}\ldots s_{p_{J}}\right],$$s_{i}\in {ℜ}^{p_{j}},$$i=1,2,\cdots p_{j}$

10.     b) Reconstruct matrix $cD_{j}$ by employing first *m *principal components $u_{1},u_{2},\ldots u_{m},$*s.t. *$\rho_{m}\geq p$

11.         $cD_{j}\leftarrow cD_{j}\times \left(I\times I^{T}\right)/p_{{}_{j}}+\overset{m}{\underset{i=1}{\sum }}u_{i}\times s_{i}^{T},$$I={\left[1,1,\cdots 1\right]}^{T}\in {ℜ}^{p_{j}}$

12.   *end if*

13. *end for*

14. **Step 3**. *Approximate the original data by the inverse discrete wavelet transform*

15. $X_{*}^{T}\leftarrow inverseDWT\left(\left[cD_{1},\;cD_{2}\cdots cD_{J};cA_{J}\right]\right)$ with the wavelet  ψ

### Tuning parameters in derivative component analysis

Although an optimal DWT level can be obtained theoretically by following the maximum entropy principle [19], it is reasonable to adaptively select the DWT level *J *according to the *'nature' *of input data, where large #samples corresponds to a relatively large *J *value, for the convenience of computation. Although the convolution in the DWT always introduces a few extra entries into each feature's corresponding detail coefficient vector in *cDj+1 *such that its length is slightly more than the half of that of in *cD_j _*[18], we have found that a large transform level does not show advantages compared with the a small transform level in feature selection. However, a small transform level (e.g., *J = 3) *may bring some hard time in separating subtle and global data characteristics because of the limited choice for the cutoff *τ*. As such, we select the DWT level as $4\leq J\leq \left\lceil {\text{log}}_{2}p\right\rceil$ considering the magnitude level of the #samples, i.e. $p\sim O\left(10^{2}\right)$ for a proteomics data set. Correspondingly, we empirically set the cutoff as 1<τ≤J/2 to separate the fine and coarse level detail coefficient matrices for its robust performance.

Furthermore, we require the wavelet  ψ in the DWT to be orthogonal and have compact supports such as *Daubechies *wavelets (e.g., '*db8*'), for the sake of the subtle data behavior capturing. The variability explanation ratio threshold is usually set as *ρ ≥ 60%*, which means the reconstructed fine level detail coefficient matrix *cD_j _*(*1 ≤ j ≤ τ*) contains at least 60% variances of the original one, to retrieve the most important subtle data behaviors interpreted by *cD_j_*. Interestingly, we have found that the first PC of each fine-level detail coefficient matrix usually count quite a high variability explanation ratio (e.g. >60%) for each fine-level detail coefficient matrix *cD_j _*(*1 ≤ j ≤ τ *). Thus, we relax the variability explanation ratio threshold ρ by only using the first PC to reconstruct each *cD_j _*matrix to catch the subtle data characteristics along the maximum variance direction. In fact, we have found that using more PCs in the fine-level detail coefficient matrix reconstruction does not demonstrate advantages in subtle data characteristics extraction and de-noising than using the first PC.

**Figure 1:**
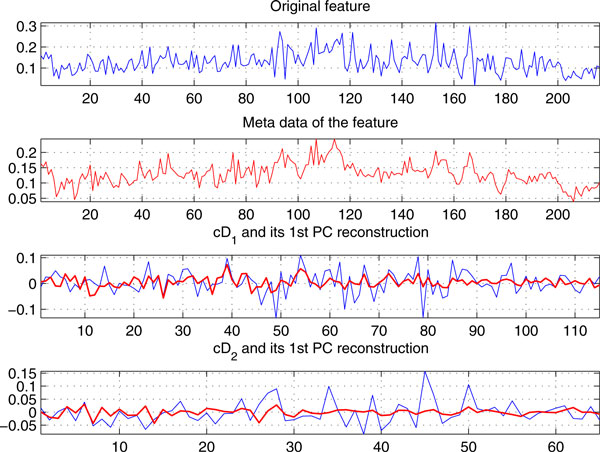
**A feature in *Ovarian-qaqc *data and its meta-data computed from DCA**. The detail coefficients cD1,cD2 (blue color) and their first PC reconstructions (red color) in DCA.

Figure 1 shows the meta-data of a feature obtained by DCA on *Ovarian-qaqc *data with 95 controls and 121 ovarian cancer samples across 15,000 *m/z *ratios [20], and its two level detail coefficient reconstructions under DCA with *τ*=*2, J *= *7*, and wavelet ***'db8'***. Interestingly, the meta-data are *smoother *and have *values in a smaller range *than the original feature for its subtle data characteristics capturing and de-noising, which reflect the true expression of the peptides/proteins at the *m/z *ratio better. In other words, DCA provides a 'zooming' mechanism to capture the original data's subtle behaviors that are usually latent in general feature selection methods. It is noted that similar results can be obtained for other mass spectral proteomic profiles also.

**Figure 2:**
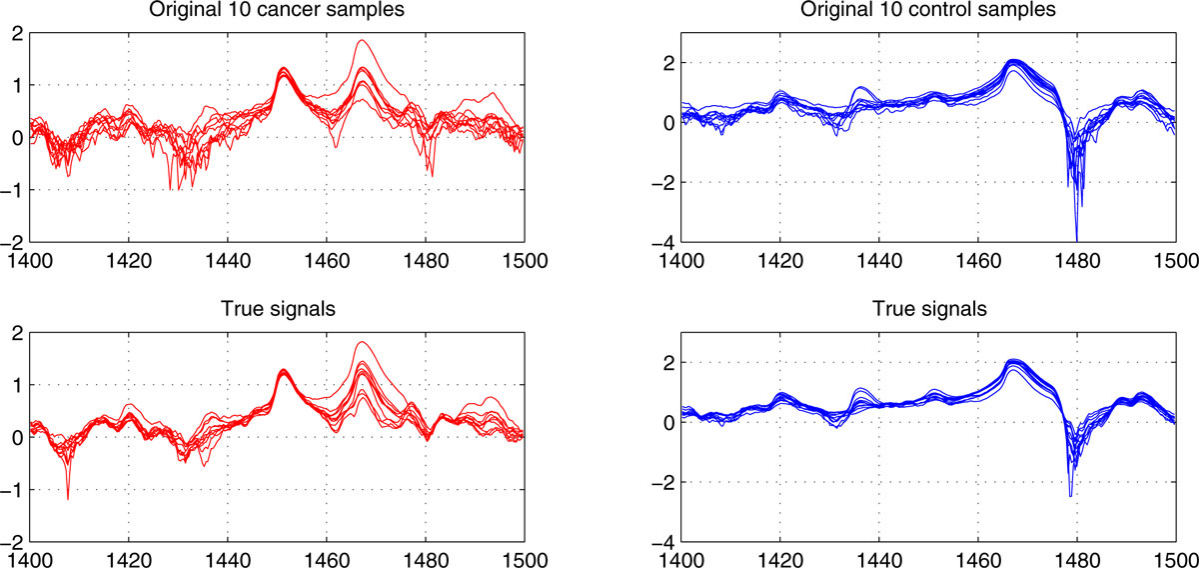
**The true signals of 10 cancer and control samples of the *Colorectal *data between 1400-1500 Da**.

In fact, the meta-data obtained from DCA can be viewed as "true signals" separated from red herrings for each serum proteomics data set. Figure 2 shows the true signals of the 10 cancer and control samples, which are randomly selected from *Colorectal *data [17] with total 48 controls and 64 cancer samples across 16,331 *m/z *ratios, extracted by our DCA under the cutoff *τ*=*2*, transform-level *J *= *7*, and wavelet *'db8'*. For the convenience of description, true signals are highlighted between 1,400 Da and 1,500 Da. Interestingly, the each type of samples in the extracted true signals appear to be smoother and more proximal to each other besides demonstrating less variations, because of major subtle data characteristics extraction and system noise removal. Obviously, from a classification viewpoint, these true signals will contribute to high accuracy diagnoses than the original proteomic data, because the built-in noises and redundant global data characteristics would have a much lower chance to get involved in classification due to derivative component analysis. Instead, subtle data characteristics would have a greater chance of participating in the decision rule inference.

### Disease diagnosis with Derivative Component Analysis

Since DCA can separate true signals from red herrings by extracting subtle data characteristics and removing built-in noises, it is natural to combine DCA with the start-of-the-art classifiers to demonstrate its effectiveness in serum proteomic disease diagnosis. We choose support vector machines (SVM) for its efficiency and popularity in translational bioinformatics [21]. As such, we propose novel derivative component analysis based support vector machines (DCA-SVM) to handle serum proteomic disease diagnosis, which is equivalent to a binary or multi-class classification problem. Thus, we briefly describe the corresponding binary and multiclass DCA-SVM as follows.

Given a binary type training samples $X={\left[x_{1},x_{2},\cdots x_{p}\right]}^{T}$ and their labels ${\left\{x_{i},c_{i}\right\}}_{i=1}^{p},$$c_{i}\in \left\{-1,1\right\}$ its corresponding meta-data $Y={\left[y_{1},y_{2},\cdots y_{p}\right]}^{T}$ are computed by using DCA, Then, a maximum-margin hyperplane: $O_{h}:w^{T}y+b=0$ in ${ℜ}^{n}$ is constructed to separate the '+1' ('cancer') and '-1' ('control') types of the samples in the meta-data *Y*, which is equivalent to solving the following quadratic programming problem (standard SVM, i.e., C-SVM):

(1) $$\begin{array}{c} {\text{min}}_{w,b,\xi }\frac{1}{2}||w|{|}_{2}^{2}+C\sum_{i=1}^{p}\xi_{i}\\ s..t.\;\;c_{i}\left(w^{T}y_{i}+b\right)\geq 1-\xi_{i},=1,2\ldots p\\ \;\;\;\;\xi_{i}\geq 0 \end{array}$$

The C-SVM can be solved by seeking the solutions to the variables $\alpha_{1}$ of the following Lagrangian dual problem,

(2) $$\begin{array}{c} {\text{max}}_{\alpha }\overset{p}{\underset{i=1}{\sum }}\alpha_{i}-\frac{1}{2}\sum_{i=1}^{p}\sum_{j=1}^{p}\alpha_{i}\alpha_{j}c_{i}c_{j}y_{i}^{T}y_{j}\\ s..t.\;\;\sum_{i=1}^{p}\alpha_{i}c_{i}=0,0\leq \alpha_{i}\leq C_{i},i=1,2,\ldots p\\ \;\;\;\;\;\;\;\;\xi_{i}\geq 0 \end{array}$$

The normal of the maximum-margin hyperplane can be calculated by the equation $w=\sum_{i=1}^{p}\alpha_{i}c_{i}y_{i},$ where the sparsity of variables $\alpha_{1}$*i = 1,2,...p*, makes classification only dependent on few training points, which are few cancerous patients or healthy subjects in the proteomics data used for training. The decision function $f\left(x^{\prime }\right)=sign(\sum_{i=1}^{p}\alpha_{i}k\left(y_{i}•y^{\prime }\right)+b$ is used to determine the class type of a testing sample $x^{\prime },$ where $y^{\prime }$ is its corresponding meta-sample computed from DCA. The function $k\left(y_{i}•y^{\prime }\right)$ is a kernel function mapping  y and $y^{\prime }$ into a same-dimensional or high-dimensional feature space. In this work, we employ the *'linear' *kernel k(x•y)=(x•y) for its simplicity and efficiency (more detailed reason for such a kernel selection can be found in the following section). Such a decision function answers the query: 'is this proteomic sample is from a patient with a specified disease or a normal individual?'

Our multiclass DCA-SVM algorithm employs the *'one-against-one' *for its proved advantage over the *'one-against-all' *and *'directed acyclic SVM' *methods [21,22]. The *'one-against-one' *method builds *k(k-1)/2 *binary SVM classifiers for a data set with *k *classes *{1,2,...k}*, each of which correspond to a pathological state. Each classifier is trained on data from two classes, i.e. training samples are from the *i-th *and *j-th *classes, *i, j = 1, 2 ... k*. After building all *k(k-1)/2 *classifiers, we employ the *'Max-wins' *voting approach to infer its final class type: if the local decision function says $x^{\prime }$ is in the class *i*, then the class *i *wins one vote; Otherwise, the class *j *wins one vote. Finally, sample $x^{\prime }$ will belong to the class with the largest vote.

### The DCA-SVM 's advantages over SVM in disease diagnosis

It is worthwhile to point out that, compared with the standard SVM, our DCA-SVM has a different feature space due to the true-signals extraction from DCA, which leads to a more robust decision rule than the standard SVM (C-SVM) for inviting the de-noised data with the subtle data characteristics in the optimal hyperplane construction. Obviously, the decision rule inferred from our DCA-SVM would avoid the traditional bias from that of the standard SVM. On the other hand, the standard SVM's feature space usually contains noises from input proteomic data, and misses the subtle data characteristics, which limit the classifier's performance and lead to a biased, global data characteristics favored decision rule.

Alternatively, the DCA-SVM 's feature space contains 'de-noised' true signals with the subtle data characteristics, which avoids the global data characteristics favored decision rule inference because the subtle data characteristics are also invited in SVM hyperplane construction besides the global data characteristics. As such, the DCA-SVM can efficiently detect those samples with similar global characteristics but different subtle characteristics in disease diagnosis than the standard SVM, which contributes to the high accuracy diagnosis.

## Results

We demonstrate our DCA-SVM can achieve rivaling-clinical diagnosis by using five benchmark high-dimensional serum proteomic data sets [17,20,23-25] and compare it with state-of-the-art peers on these data. We introduce details about the data sets as follows.

### Data sets

The benchmark data sets used in the experiment are heterogeneous data generated from different experiments via different high-resolution serum profiling technologies such as MALDI (matrix-assisted laser desorption)-TOF (time-of-flight), SELDI (surface enhanced laser desorption and ionization)-TOF (time-of-flight), and SELDI-QqTOF (quadrupole time-of-flight). The details of the data sets are as follows.

*Cirrhosis *data set is a three-class MALDI-TOF serum proteomic data with total 201 spectra that consisting of 72 samples from healthy individuals, 78 samples from patients with hepatocellular carcinoma (HCC), the most common liver cancer, and 51 samples form cirrhosis patients, across 23,846 *m/z *ratios [24]. As the major cause of hepatocellular carcinoma, cirrhosis can be viewed as a key intermediate stage pathologically between a normal state and a state with hepatocellular carcinoma.

*Colorectal *(CRC) data set consists of 48 control and 64 cancer spectra across 16,331 *m/z *values [17], which are selected from the raw data with 65, 400 *m/z *values profiled by MALDI-TOF technologies to cover a range from 0.96 to 11.16 kDa; *HCC *data set is a binary SELDI-QqTOF proteomic data with total 358 spectra that consisting of 181 controls and 176 cancers across 6,107 *m/z *ratios, which are selected from about 340,000 *m/z *values through a binning procedure for original mass spectra [23]. As a well-known benchmark data, *Ovarian-qaqc *data consist of 95 controls and 121 ovarian cancers across 15,000 *m/z *values, which is a high-resolution serum proteomics data produced by SELDI-TOF profiling [20]. *Toxpath *data were generated from a toxicoproteomics experiment to conduct serum proteomic diagnosis for doxorubicin-induced cardiotoxicity by Petricoin et al [25]. This data set has 115 mass spectra consisting of 28 normal, 43 potential normal, 34 cardiotoxicities, and 10 potential cardiotoxicities, across 7,105 *m/z *values, which were obtained by a binning procedure from ~350,000 *m/z *values in the raw data.

It is worthwhile to point out that these data sets are preprocessed by different methods. In fact, we conducted baseline correction, smoothing, normalization, and peak alignment for the *Ovarian-qaqc *data. The baseline for each profile was estimated within multiple shifted windows of widths 200 *m/z*, and the spline approximation was employed to predict the baseline. The mass spectra were further smoothed using the '*lowess*' method, and normalized by standardizing the area under the curve (AUC) to the group median [26]. Alternatively, we only conducted the baseline correction, normalization and smoothing for the *HCC *and *Cirrhosis, HCC *and *ToxPath *data (The smoothing method is selected as a different '*least-square polynomial' *algorithm) [25,26]. We did not conduct our own preprocessing for the *Colorectal *data because it was preprocessed data [17]. Table 1 sketches the basic information about the five mass spectra data.

**Table 1:** Benchmark proteomic data


**Data**	**#Feature**	**#Sample**	**Platform**

*Cirrhosis*	23846	72 controls + 78 HCCs + 51 cirrhosis	MALDI-TOF
*Colorectal*	16331	48 controls + 64 cancers	MALDI-TOF
*HCC*	6107	181 controls +176 cancers	SELDI-QqTOF
*Ovarian-qaqc*	15000	95 controls + 121 cancers	SELDI-TOF
*ToxPath*	7105	28 normals + 43 potential normals + 34 cardiotoxicities + 10 potential cardiotoxicities	SELDI-QqTOF

### The state-of-the-art comparison algorithms in proteomic diagnosis

We compare our DCA-SVM based profile biomarker diagnosis with following state-of-the-arts in this work. They include a partial least square (PLS) based linear logistic discriminant analysis (PLS-LLD) [27,28], standard SVM [21], a SVM combining with principal component analysis: PCA-SVM [8], and a SVM with input-space feature selection: *fs*-SVM.

These comparison classifiers can be categorized into three groups, i.e., The group 1 only consists of standard SVM itself; The group 2 consists of those classifiers integrating SVM with input space and subspace feature selection methods respectively, i.e., PCA-SVM and *fs*-SVM; The group 3 consists of a non-SVM classifier, which employs partial least square (PLS) to conduct dimension reduction for linear logistic discriminant analysis [27,28]. The reason we select PLS-LLD classifier is that it generally outperforms the other similar non-SVM (e.g., PCA-LDA) methods according to our implementations and Sampson et al 's work [29].

It is noted that we employ two different input-space methods: *t-test *and *anona1 (one-way ANOVA) *in *fs*-SVM to conduct feature selection for binary and multi-class data respectively [30]. Since serum proteomics data usually follow or approximately follow a normal distribution after normalization, it is reasonable to use a two-sample *t-test *to rank each feature under a binary case. For multi-class data such as *Cirrhosis *and *Toxpath*, we use one-way *ANOVA (anova1) *to identify its statistically significant features [30]. As such, we select a feature set including all features with *p-values < 0.05 *under *the t-test *and *anova1 *for each data. Moreover, since the PLS-LLD classifier involved matrix inverse calculation, which is notorious for its high computing demand for a large matrix (e.g., a 5,000 × 5,000 matrix), we only pick 2000 top-ranked features from for this method to avoid large computing overhead.

#### Kernel selection, cross validation, and parameter setting

It is noted that we employ the *'linear' *kernel k(x,y)=(x•y) in all SVM-related classifiers for its efficiency in omics data classification, rather than nonlinear kernels (e.g., Gaussian kernels). In our previous work, we actually have pointed out that nonlinear kernels (e.g., Gaussian kernels) would lead to overfitting for gene expression and proteomics data [6,8]. Although Gaussian kernels are quite popular in serum proteomics diagnosis, it would give deceptive diagnosis due to overfitting [6]. In fact, we will show serum proteomics data diagnosis is a linear separable problem, for which a linear kernel should be the optimal kernel selection in next section.

To avoid potential biases from presetting training/test data partition on classification, we employ the *k*-fold (k = 5) cross-validation in our experiments to evaluate the five classifiers' performance for all data sets instead of the independent test set approach. In the 5-fold cross-validation, proteomic samples are randomly partitioned into k = 5 folds equally, k = 4 folds are used as training data each time, the fold left is used for evaluation. Such a process is repeated k = 5 times. In addition to choosing the first ten PLS components in the PLS-LLD classifier, we uniformly set the transform level *J = 7*; cutoff *τ = 2*; and apply the first loading vector based detail coefficient matrix reconstruction in DCA for all data sets for the convenience of comparison, though these parameter setting may not be optimal.

#### Diagnostic performance measures

Before we demonstrate our profile biomarker approach's advantages. We introduce several key diagnosis performance measures, which are diagnostic accuracy, sensitivity, specificity and positive predication ratios, as follows. The diagnostic accuracy is the ratio of the correctly classified test samples over total test samples. The sensitivity, specificity, and positive predication ratio are defined as the rates *TP/(TP+FN), TN/(TN+FP)*, and *TP/(FP+TP) *respectively, where *TP (TN) *is the number of positive (negative) targets (a positive (negative) target is a proteomic sample with '+1' ('-1') label) correctly diagnosed and *FP (FN) *is the number of negative (positive) targets incorrectly diagnosed by the classifier (e.g., SVM). It is noted that the sensitivity, specificity, and positive predication ratio for multiclass data *Cirrhosis *and *Toxpath *are obtained by treating them as a corresponding binary data. For instance, we group 78 *HCC *and 51 c*irrhosis *samples into a same class type.

**Figure 3:**
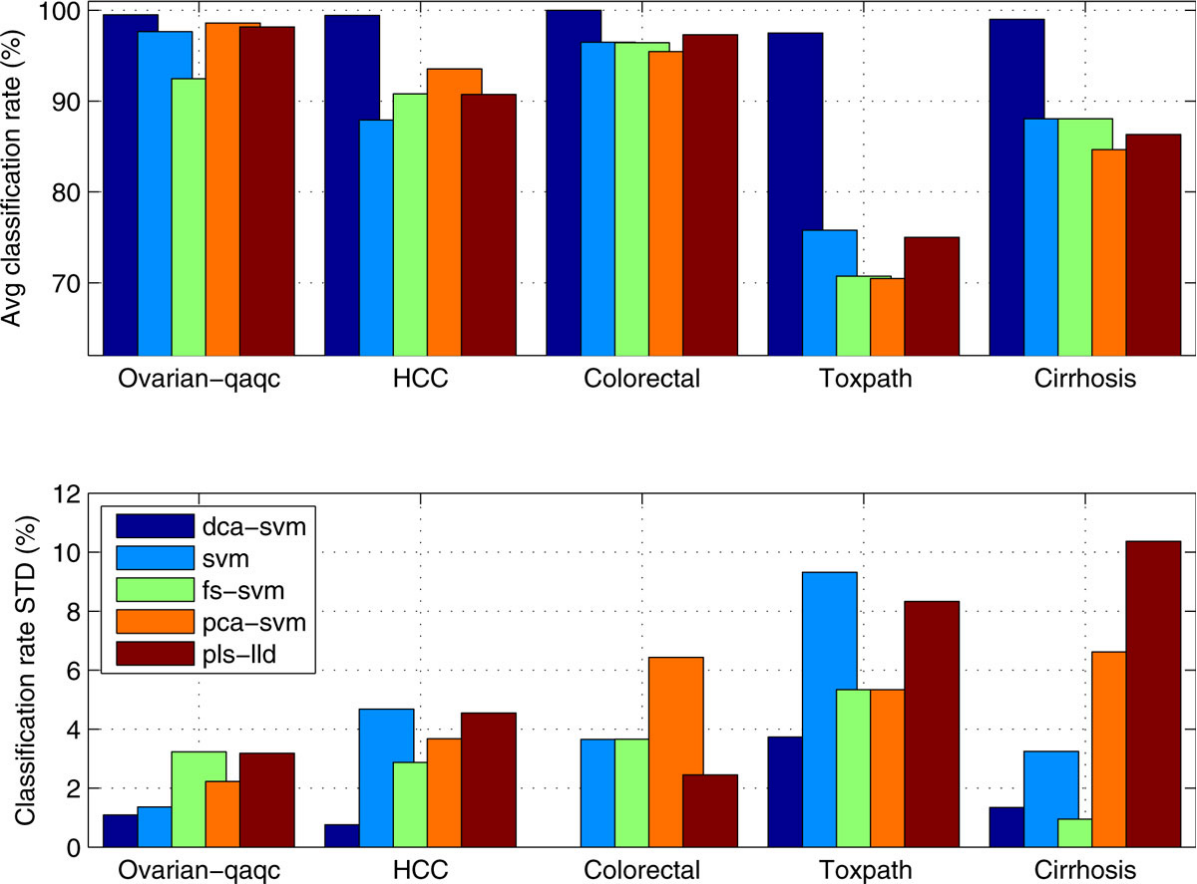
**Comparing DCA-SVM based profile-biomarker diagnosis' average diagnostic accuracies and its standard deviations with those of other peers**.

Figure 3 compares the DCA-SVM's average diagnosis and its standard deviations with those of the comparison algorithms. We have found that proposed DCA-SVM achieves a nearly rivaling-clinical level diagnosis and demonstrates strongly leading advantages over its peers in a stable manner. Alternatively, those comparison algorithms seem to show quite large level oscillations that indicate that the classifiers lack stability and good generalization capacities across different data sets, which probably exclude themselves as candidates for clinical proteomics diagnosis.

For example, DCA-SVM achieves 99.52% (sensitivity: 100%, specificity: 99.17%), 100% (sensitivity: 100%, specificity: 100%), and 99.44% (sensitivity: 98.00%, specificity: 100%) diagnostic accuracies on the *Ovarian-qaqc, Colorectal *and *HCC *data respectively. However, the SVM classifier only attains corresponding 97.68% (sensitivity: 96.78%, specificity: 98.40%), 96.48% (sensitivity: 96.92%, specificity: 95.78%), 87.93% (sensitivity: 90.32%, specificity: 85.62%) diagnostic accuracies respectively for these three data sets.

Such a consistently leading performance is highlighted further in multiclass phenotype diagnosis. Our DCA-SVM algorithm reaches 97.50%, 99.01% diagnostic rates for *Toxpath *and *Cirrhosis *data respectively. However, the SVM classifier can only achieve 75.80% and 88.06% diagnosis for the same data sets respectively.

Although the input-space or subspace methods may boost diagnosis sometimes for binary-type data set (e.g., for *HCC *data PCA-SVM, *fs*-SVM attains 93.56% and 90.18% diagnosis which are higher than the 87.93% diagnostic ration from the SVM classifier), they seem not be able to increase a SVM classifier's diagnosis and generation abilities significantly, especially for multiclass data. For instance, the *fs*-SVM and PCA-SVM both have lower or the same level diagnosis than the original SVM without feature selection on *Toxpath *and *Cirrhosis *data. This may suggest the selected features' unpredictable impacts on serum proteomics diagnosis due to the input and subspace feature selection methods' limitations in de-noising and latent data characteristics capturing.

In contrast to the proposed DCA-SVM algorithm, all the comparison algorithms including PLS-LLD, which achieves slightly better diagnosis than SVM, PCA-SVM, and *fs*-SVM, shows high-level oscillations in diagnosis like the others, across different data. It is noteworthy that the high-level oscillations in diagnosis is further highlighted by corresponding large standard deviation values in diagnosis from those classifiers in Figure 3, where DCA-SVM demonstrates its good stability and generalization for its smallest standard deviation values across all the data sets.

We have to point out that such an excellent performance is because DCA forces the SVM hyperplane construction to rely on the both latent and global data characteristics in a de-noised feature space under a linear kernel, which contributes to a robust and consistent high-accuracy diagnosis. Such consistent performance applies all five data sets, which prevents from any possible overfitting possibility. On the other hand, just as we pointed out in our previous work, overfitting always happens on nonlinear kernels (e.g., Gaussian kernels) in omics data classification [6,8].

#### A potential solution to overcome the data reproducibility

**Figure 4:**
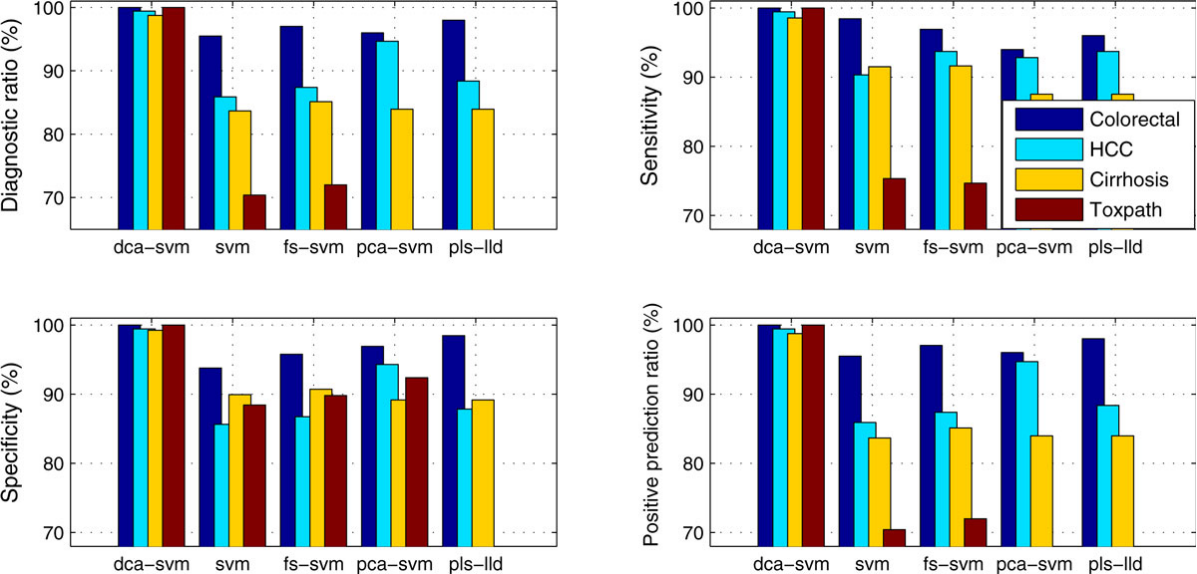
**Comparing profile-biomarker diagnosis' diagnostic accuracies, sensitivity, specificity, and positive predication ratio with those of other peers across four proteomics data under 5-fold cross validation**.

Figure 4 compares the performance of five classifiers across four data sets under k-fold (k = 5) cross validation in terms of diagnostic accuracy, sensitivity, specificity and positive predication ratios. It seems that DCA-SVM has attained strong advantages over its peers in terms of diagnostic measures. In fact, all classifiers except DCA-SVM show relatively high-level oscillations for these diagnostic measures. For example, *fs*-SVM achieves 96.48% diagnosis for the *Colorectal *data but only 70.47% for the *Toxpath *data. To further demonstrate DCA'superiority in serum proteomics data diagnosis, we compare DCA-SVM results with those previous results obtained for these data sets in the literature as follows.

For *Colorectal *data, a 97.5% diagnosis accuracy with sensitivity 98.4% and specificity 95.8% were attained under 5-fold cross-validation in [17], where a wavelet transform is directly applied to each proteomic sample by applying Kolmogorov-Smirnov (KS) and Mann-Whitney (MW) tests to the wavelet coefficients before calling a standard SVM classifier [30]. However, our DCA-SVM achieves 100% diagnosis accuracy with sensitivity 100% and specificity 100%. It is worthwhile to point out that our comparison algorithms: *fs*-SVM and PLS-LLD have attained 96.48% (sensitivity: 96.92%, specificity: 95.78%), and 97.31% (sensitivity: 96.00%, specificity: 98.46%) diagnosis accuracies with very general feature selection under 5-fold CV ([14] uses a double CV consisting of 5-fold CV and leave-one-out CV).

For *HCC *data, a ~90%+ diagnosis accuracy with sensitivity 91% and specificity 92% is achieved by a particle swarm optimization based support vector machines (PSO-SVM) with baseline selection under a 10-fold cross-validation [23]. Instead, our DCA-SVM achieves 99.44% diagnosis accuracy (sensitivity: 99.44%, specificity: 99.44%) under 5-fold CV. In fact, all comparison algorithms expect SVM achieves same or high level performance than the previous PSO-SVM approach.

For *Ovarian-qaqc *data, our DCA-SVM achieves a 99.53% clinical-level diagnosis accuracy with sensitivity 98.95% and specificity 100%, which is better than the original diagnosis level obtained in [23] and all the other peers; For *Cirrhosis *data, Ressom *et al *partitioned this three-class data into two binary data sets and proposed a novel hybrid ant colony optimization based support vector machines (ACO-SVM) to achieve 94% and 100% specificity to distinguish hepatocellular carcinoma (HCC) from *Cirrhosis *[24]. There was no result available to distinguish normal, HCC, and cirrhosis in a multiclass diagnostic way. However, our proposed DCA-SVM has achieved 99.01% diagnosis accuracy for this multi-class data sets; The DCA-SVM achieves a rivaling clinical diagnosis accuracy 97.5% for the *Toxpath *data, which is a subset of the original data with 203 samples in [25] (we remove the 88 samples whose class-type is 'unknown' to avoid ambiguity in diagnosis).

It is noted that those algorithms applied to these data sets are generally individualized methods designed for a specific proteomics data. However, our proposed derivative component analysis based classifier (DCA-SVM) can apply to all data sets generated from different experiments and profiling technologies with rival-clinical diagnosis. Moreover, since DCA outputs a same-dimensional meta-data for each input proteomics data, it seems to be able to provide a potential profile-biomarker approach to overcome the data reproducibility issue by viewing the meta data as a uniform profile-biomarker by employing DCA-SVM to achieve rivaling-clinical diagnosis. To some degree, DCA and DCA-SVM show some promising to use a profile-biomarker way to resolve such a problem for its latent data characteristics extraction and exceptional diagnosis.

### Serum proteomics data are linearly separable

Our DCA-SVM algorithm's rivaling clinical level performance may suggest that serum proteomic data classification can be a linearly separable problem under appropriate feature selection. Such a proposition would provide a direct theoretical support to clarify some doubts about the nonlinearity in serum proteomics data may prevent it from complex disease diagnosis clinical routine [3,5,17], and suggest feasibility to conduct disease phenotype discrimination by using few biomarkers. In other words, if serum proteomics data are linearly separable, then, using biomarker patterns can guarantee disease phenotype discrimination, which is a key in early cancer discovery. Otherwise, seeking biomarker patterns only have a partial meaning if serum proteomics data are linearly non-separable or nonlinear because these biomarkers cannot attain 100% or rival clinical (e.g., 99%) disease phenotype separation. Moreover, serum proteomics data are linearly separable indicates '*linear *' kernels rather than nonlinear ones would be optimal one for SVM in disease diagnosis. We sketch the definition of a linear separable problem as follows.

#### Linearly separable problem

A linearly separable problem can be simply described as follows. Given $P={\left[x_{1},x_{2},\cdots x_{N}\right]}^{T},$$Q={\left[y_{1},y_{2},\cdots y_{M}\right]}^{T},$$x_{i}y_{j}\in {ℜ}^{n},$i=1,2,⋯N,j=1,2,⋯M, if there exists a hyperplane *H: *$w^{T}v+b=0,$$w,v,\in {ℜ}^{n},$b∈ℜ, such that ∀x∈P,∀y∈Q,$w^{T}x+b>0$ and $w^{T}y+b<0,$ then *P *and *Q *are linearly separable data, i.e. classifying P and Q is a linearly separable problem. In other words, it is equivalent to mapping entries in *P *and *Q *to two different types of labels (e.g., +1 and -1) respectively. Such a definition can be extended similarly to more than two sets, e.g., *P_1_; P_2_*...*P_m_*, *m ≥ 2*, which is equivalent to mapping the *m *sets to the labels *1,2,...m *respectively.

It's clear to see that binary and multiclass SVMs by nature are linear separable test methods for its optimal hyperplane construction. However, due to the fact that serum proteomic profiles are noisy data with redundant information, it is rather difficult to draw a conclusion that they are linearly separable data because of its relatively low classification accuracies from most SVM classifier.

However, the DCA-SVM's exceptional performance reaches 99.53% 99.44%, 100%, for *Ovarian-qaqc, HCC*, and *Colorectal*, respectively, which strongly demonstrates they are linearly separable data. Although DCA-SVM only achieves 97.50% and 99.01% for *Toxpath *and *Cirrhosis *respectively, which are much better than those of the state-of-the-arts, we still believe the performances indicate these serum proteomic data are linearly separable, considering possible factors to lead to small misclassifications such as complexities of multi-class SVM hyperplane construction, possible numerical artifacts in SVM algorithm implementations, and small likelihoods that the SVM decision function may not provide a deterministic answer [21]. Thus, DCA-SVM disease classification results demonstrate that these high-dimensional data are actually linearly separable in a de-noised feature space when their latent data characteristics are extracted by DCA. Alternatively, it means the linear kernel is the optimal kernel for SVM.

### DCA-MRAK: a DCA-induced biomarker discovery

Motivated by DCA-SVM's exceptional performance, we present a DCA-induced biomarker discovery algorithm: DCA-MARK to further validate the linear separability of serum proteomics data, where each biomarker can be viewed as a statistically significant feature with respect to the others [30]. That is, we demonstrate a serum proteomic data set 's linear separability by employing the few biomarkers discovered from its meta data obtained from DCA. We will demonstrate that these biomarkers from DCA-MARK can easily separate disease phenotype completely for high-dimensional proteomics data. To the best of our knowledge, there is no similar result available in the previous research. The *DCA-MARK *can be sketched as follows.

1). Given an input dataset $X\in {ℜ}^{n\times p},$ we seek the biomarkers by looking at its meta data $X^{*}$ from DCA through scoring and ranking each feature in $X^{*}$ by using the *t*-statistic for the binary data and *F*-statistic for the multiclass data [30].

2). Given a feature in a binary-class dataset $x=x_{1}\cdots x_{n_{1}+1}\cdots y_{n_{1}+n_{2}}$ in $X^{*},$ the *t-statistic *is calculated as $t=|\bar{x}-\bar{y}|/\sqrt{s_{x}^{2}/n_{1}+s_{y}^{2}/n_{2}},$ where $\bar{x},\bar{y},$$s_{x}^{2},s_{y}^{2}$ are the mean and variance values of the two classes of entries in the feature *x*. In practice, we can employ the pooled variance estimation to calculate a same variance for two types of entries as $s_{p}^{2}=\left(\left(n_{1}-1\right)s_{x}^{2}+\left(n_{2}-1\right)s_{y}^{2}\right)/\left(n_{1}+n_{2}-2\right).$

3). Given a feature in a multi-class dataset with *k > 2 *classes, the *F-statistic *is calculated as $F=\sum_{j=1}^{k}{n_{j}/\left(\bar{x_{j}^{*}}-\bar{\bar{x^{*}}}\right)}^{2}/(k-1))/\sum_{j=1}^{k}\left(n_{j}-1\right)s_{j}^{2}/\left(n_{T}-k\right)),$ where $n_{j}$ is the sample size, parameters $x_{j}^{*}$ and $s_{j}^{2}$ are the sample mean and sample variance for the *j-th *class. $\bar{\bar{x^{*}}}=\sum_{j=1}^{k}\sum_{i=1}^{n_{j}}x_{ij}^{*}/n_{T}$is the overall sample mean where $x_{ij}^{*}$ is the expression value of *i-th *observation for the class *j *and $n_{T}=\sum_{j=1}^{T}n_{j}$ is the total sample size for the *k *groups.

4). The biomarkers are the top-ranked features with the largest statistic values or the smallest *p-values*, i.e. we pick the three top-scored biomarkers for the sake of 3-dimensional visualization convenience.

**Figure 5:**
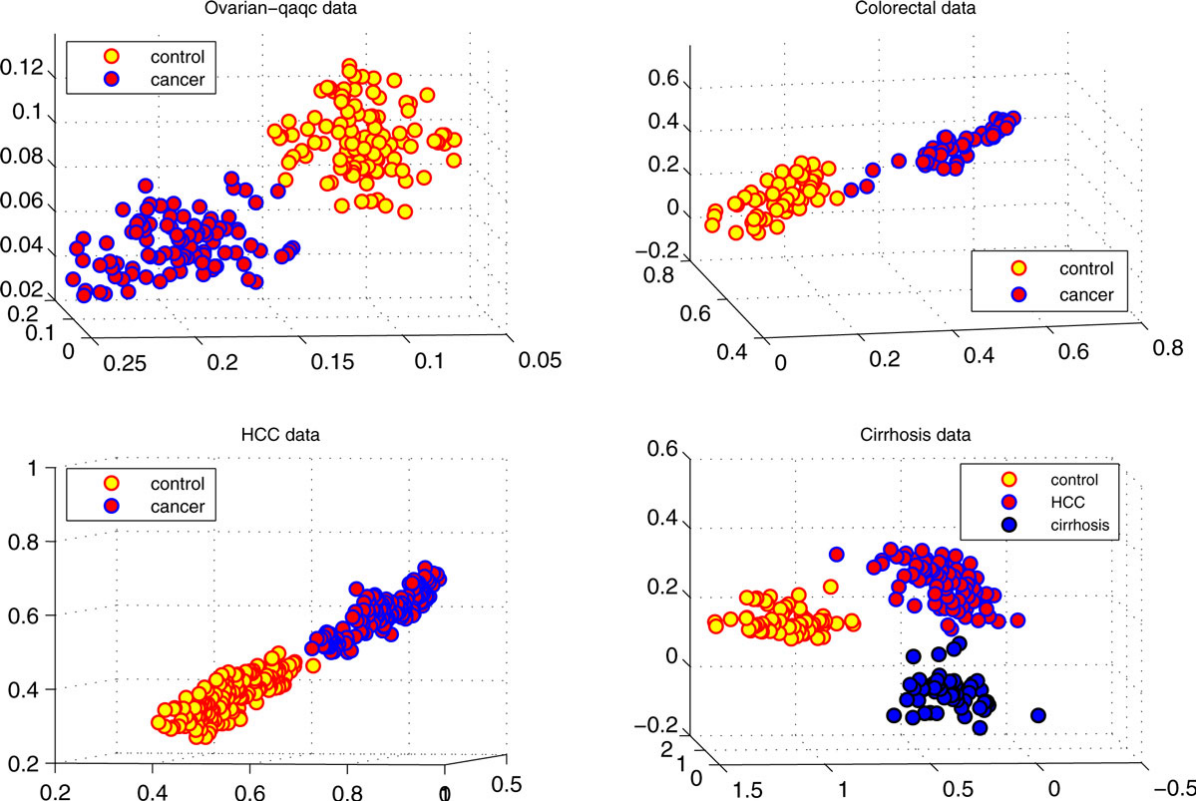
**Separating disease phenotypes of four serum proteomic data sets by only using their three biomarkers with the smallest p-values**.

Figure 5 illustrates the separation of four benchmark data sets with three top-ranked biomarkers (peaks) from DCA-MARK. It is interesting to see that these high-dimensional proteomic profiles can be separated almost completely with few biomarkers identified from DCA-MARK. We can also obtain meaningful biological depth by checking these biomarkers. For example, the SW plot in Figure 5 shows the separation of 176 controls and 181 cancers in the *HCC *data, which is generated by high resolution mass spectral SELDI-Qq-TOF platform, by the top-ranked biomarkers (peaks) at 2534.2, 2584.3, and 6486.2 *m/z *ratios, where each dot represents a sample (a patient with HCC or a healthy subject). It is clear that we achieve linear separability for this data by using only three biomarkers. It is also interesting to see that two biomarkers are from downstream *m/z *ratios, which were believed to be more sensitive to detect phenotype information than those from upstream *m/z *ratios [24].

Such a separation actual fits to the linearly separable case for an SVM classifier. Thus, it is quite easy to identify a hyperplane to separate two classes phenotypes completely. For example, we run SVM for the three biomarkers for the total 357 samples and achieve 100% classification accuracy (sensitivity: 100%, specificity: 100%). Such a result demonstrates a strong advantages in phenotype discrimination over the previous work [17,23,24], just as we pointed out before, which employed quite complicate evolutionary algorithm (PSO-SVM) to collect a set of informative peaks and achieved 90%+ diagnosis accuracy under a 5-fold cross validation [23].

Moreover, we select three top-ranked biomarkers at 1668.99, 5907.73, 5907.13 m/z ratios for the *Cirrhosis *dataset, which is a three-class high-resolution MALDI-TOF proteomic profile with 23,846 features [24]. In addition to demonstrating the linear separability, the phenotype separations provided by the three biomarkers give very meaningful biological information. The SE plot in Figure 5 shows the three clearly separable clusters, where Cirrhosis cluster with 51 samples (blue) have closer spatial distances to the HCC cluster 78 samples (red) than the normal cluster with 72 samples (yellow). Such spatial distances demonstrated by our biomarkers are actually consistent to their pathological distances: Cirrhosis is the middle stage to hepatocellular carcinoma (HCC) for a healthy subject [31]. To the best of our knowledge, no previous work achieved the similar results.

## Discussion

In this study, we propose a novel feature selection algorithm: derivative component analysis (DCA) to overcome the weakness of the traditional feature selection methods. Unlike the traditional methods, the DCA focuses on latent data characteristics gleaning and de-noising by analyzing derivative data components for input data to calculate a same dimensional meta-data.

We further embed derivative component analysis into support vector machines to achieve rivaling clinical level phenotype discrimination for five benchmark serum proteomics data by comparing it with the other state-of-the-arts. The DCA-SVM 's exceptional classification accuracies suggest the serum proteomics data's linear separability and further inspire DCA-MARK, a DCA-induced biomarker discovery approach, which in turn demonstrate high-dimensional proteomics data 's linear separability with few biomarkers. Moreover, derivative component analysis (DCA) demonstrate a potential to resolve data reproducibility problem of serum proteomics by viewing each input data's meta-data as a profile biomarker by employing DCA-SVM to achieve clinical level disease diagnosis, because of DCA's true signal extraction for input proteomics data.

Such profile biomarker diagnosis approach actually demonstrates strong advantages over the existing biomarker discovery oriented diagnosis by treating input proteomic data as a profile biomarker. The systems approach seems to fit the "personalized diagnostics" better [32], because it can be difficult both biologically and computationally to achieve a clinical level diagnostics for those complex diseases like cancer, in which thousands genes can be involved, based on several differentially expressed proteins, especially when the source data suffer from the reproducibility issue.

Our experimental results demonstrated that the DCA's parametric tuning works efficiently though they may not be the optimal ones theoretically. It is possible to seek optimally parametric settings in derivative component analysis for each proteomic data from an information entropy analysis or Monte Carlo simulation standing point [18]. However, we are not sure such computing demand way is practically worthwhile because the clinical level diagnostics are already attained under our current parametric tuning.

## Conclusions

Our DCA provides an alternative feature selection by implicitly extracting useful data characteristics whiling maintaining the data 's original dimensionality. It suggests that subtle data characteristics gleaning and de-noising may be more important in proteomics data feature selection and following phenotype discrimination. It is worthwhile to point out that DCA-related techniques developed can be also applied to gene expression data smoothly. Although we are quite optimistic to see that our DCA-MARK can capture meaningful peaks from low-weight sera from different data sets, there is still an urgent need to verify and compare these biomarkers with the previous ones to seek potential pathological meaning and clinical application. Although derivative component analysis does show a potential to conquer the reproducibility problem of serum proteomics, a future concrete proteomics clinical test is still needed to explore such a potential. Although we are quite optimistic to see that our DCA-SVM based diagnosis will be a potential candidate to achieve a clinical disease diagnosis in proteomics by conquering the reproducibility problem, rigorous proteomics clinical tests are needed urgently to explore such a potential and validate its clinical effectiveness. In our ongoing work, we are working with pathologists to investigate extending the profile-biomarker diagnosis approach to TCGA and RNA-Seq data besides genes expression array analysis [33,34].

## Competing interests

The author declares that they have no competing interests.

## Authors' contributions

HAN did all the work for this paper.

## Declarations

Publication for this article has been funded by Fordham University. The author would like to thank Fordham University for awarding him faculty research fellowship to support this project.

This article has been published as part of *BMC Medical Genomics *Volume 7 Supplement 1, 2014: Selected articles from the 3rd Translational Bioinformatics Conference (TBC/ISCB-Asia 2013). The full contents of the supplement are available online at http://www.biomedcentral.com/bmcmedgenomics/supplements/7/S1.
